# HIV Infection of Macrophages: Implications for Pathogenesis and Cure

**DOI:** 10.20411/pai.v2i2.204

**Published:** 2017-05-24

**Authors:** Kiera L. Clayton, J. Victor Garcia, Janice E. Clements, Bruce D. Walker

**Affiliations:** 1 Ragon Institute of MGH, MIT and Harvard, Cambridge, Massachusetts; 2 Division of Infectious Diseases, Center for AIDS Research (CFAR), University of North Carolina at Chapel Hill (UNC), School of Medicine, Chapel Hill, North Carolina; 3 Department of Molecular and Comparative Pathobiology, Johns Hopkins University School of Medicine, Baltimore, Maryland; 4 Department of Pathology, Johns Hopkins University School of Medicine, Baltimore, Maryland; 5 Department of Neurology, Johns Hopkins University School of Medicine, Baltimore, Maryland; 6 Howard Hughes Medical Institute, Chevy Chase, Maryland; 7 Institute of Medical Engineering and Sciences, Massachusetts Institute of Technology, Cambridge, Massachusetts

**Keywords:** HIV/SIV pathogenesis, macrophages, viral reservoir, replication competent virus, animal models of HIV infection

## Abstract

Although CD4^+^ T cells represent the major reservoir of persistent HIV and SIV infection, accumulating evidence suggests that macrophages also contribute. However, investigations of the role of macrophages are often underrepresented at HIV pathogenesis and cure meetings. This was the impetus for a scientific workshop dedicated to this area of study, held in Cambridge, MA in January 2017. The workshop brought together experts in the fields of HIV/SIV immunology and virology, macrophage biology and immunology, and animal models of HIV/SIV infection to discuss the role of macrophages as a physiologically relevant viral reservoir, and the implications of macrophage infection for HIV pathogenesis and strategies for cure. While still controversial, there is an emerging theory that infected macrophages likely persist in the setting of combination antiretroviral therapy. These macrophages could then drive persistent inflammation and contribute to the viral reservoir, which indicates the importance of addressing macrophages as well as CD4^+^ T cells with future therapeutic strategies.

Despite dramatic advances that have made HIV infection a treatable disease for those fortunate enough to have access to combination antiretroviral therapy (cART), the viral infection persists. Persistent inflammation remains a problem despite cART, driving ongoing disease pathogenesis, and the prospect of life-long therapy due to persistent viral reservoirs is a medical, economic, and social challenge. These factors have led to major research efforts in the HIV/SIV research fields dedicated to establishing a cure, and to defining ways to circumvent the persistent immune activation. In both settings, major questions persist as to the potential role of macrophage infection in contributing to inflammation and reservoir persistence. This workshop was convened with leading experts in the field to address these issues.

## INTRODUCTORY SESSION

The major focus of the workshop was the interaction of HIV/SIV with macrophages, reservoir persistence, immune evasion, and the development of new technologies to characterize infected macrophages *ex vivo* and *in vivo*. The initial keynote talk by Dr. Filip Swirski (MGH/Harvard Medical School, MA) presented a stimulating overview of ontogenic and environmental control of macrophage function. Highlighting recent work, he discussed the origins of macrophages, describing the differences and similarities of long-lived, embryonically derived macrophages and short-lived, monocyte-derived macrophages (MDMs). His work showed that individual tissues accommodate different proportions of both macrophage types [1]. For example, the majority of the Kupffer cells in the liver comprise long-lived macrophages. However, during periods of stress, MDMs are recruited and become phenotypically (but not transcriptionally) indistinguishable from the embryonically derived macrophages. When the stress is resolved, the MDMs die, while the embryonically derived macrophages persist. Furthermore, some macrophages, while initially thought of as terminally differentiated, can also self-renew [2]. Such work emphasizes the need to further study the HIV/SIV reservoir beyond traditional MDM cultures, and focus on the contributions of long-lived, short-lived, and/or self-renewing macrophages to the HIV reservoir in humans.

This talk was followed by plenary speaker, Dr. Dan Kuritzkes (Brigham and Women's Hospital, MA), who discussed “Clinical Trials of HIV Cure: Where Have We Been? Where Are We Going?” providing an overview of current cure-based research, which was also highlighted at the NIH/ NIAID-sponsored “Strategies for a Cure” meeting in November 2016 (Bethesda, MD). Starting with the central dogma of HIV Cure, he summarized the strategies to reactivate and eliminate the latent reservoir [3]. While latency-reversing agents (LRAs) were discussed as methods to reactivate the reservoir, therapeutic vaccines, chimeric antigen receptor (CAR) T cells, checkpoint blockades, and immunologic agonists to harness the immune system were discussed as strategies for elimination [4]. Dr. Kuritzkes reviewed current and past cure-oriented clinical trails. Initial attempts to replicate the long-term treatment-free remission experienced by the Berlin patient — either by transplantation of allogeneic stem cells that are deleted in CCR5 or by gene therapy approaches — have as yet been met with limited success. Second, LRAs have only exhibited modest, if any reactivation of the latent HIV reservoir *in vivo*. Third, although checkpoint blockade is in its infancy, anti-PD-L1 therapy has shown promise in enhancing CD8^+^ T cell responses in HIV^+^ individuals; however, as in the field of cancer treatment, immunomodulatory therapies are limited by toxicities. Fourth, similar to immunomodulatory drugs, therapeutic vaccines have elicited favorable immunogenicity, but failed to control viremia post-antiretroviral treatment interruption (ATI). Finally, Dr. Kuritzkes highlighted a trial using passive transfer of a single broadly neutralizing antibody (BNAb) to control viral load post ATI; however, this therapy had a modest delay in time to viral rebound and no demonstrable effect on viral reservoir [5]. Thus, to date, no treatment has yielded control of viremia in the absence of cART, the minimum requirement for the patient to achieve “functional cure” status. Interestingly, none of these strategies have specifically addressed macrophages as a physiologically relevant, targetable reservoir. However, Dr. Kuritzkes' presentation, with a focus on CD4^+^ T cell reservoirs, set the stage for goals to determine the role of macrophages as a potential viral reservoir and to identify effective strategies that can be developed to target this population.

Dr. Steve Deeks (University of California, San Francisco, CA) presented the final talk of the introductory session. Providing a basic science overview of the CD4^+^ T cell reservoir, he discussed: (1) the size and stability of the reservoir, (2) T cell subsets that comprise the reservoir, (3) the source of virus production, (4) the source of viral persistence, (5) the timing of reservoir establishment, and (6) current thoughts about macrophages as a reservoir, and prevention of infection and latency reversal in this population [6–10]. Currently, sampling issues limit the study of the human reservoir, and in addition to blood, gut, and lymph nodes, more tissues will need to be sampled to achieve improved coverage. This is a major challenge for the study of infected macrophages in HIV^+^ individuals. Furthermore, although long-lived memory CD4^+^ T cells contribute to long-term persistence of the lymphocyte reservoir, the turnover rate and the spread of this lymphocyte population during cART are not completely characterized. As embryonically derived macrophages are long-lived and self-renewing, infection of these macrophages is likely to contribute to HIV persistence. Finally, Dr. Deeks emphasized that while the human macrophage contribution to the HIV reservoir requires further investigation, monocytes and macrophages clearly contribute to chronic inflammation, especially in the brain, in HIV-infected individuals. Thus, while strategies are needed to target the macrophage reservoir, treatments are also needed to resolve chronic innate inflammation to lower the incidence of non-AIDS related co-morbidities.

## SESSION #1: ESTABLISHMENT OF INFECTION IN MACROPHAGES

Dr. Ronald Swanstrom (University of North Carolina at Chapel Hill, NC), started the first session with his talk entitled “HIV-1 Is Typically R5 T Cell-Tropic: Biological Implications”. His studies suggest that 95% of worldwide HIV-1 isolates use CCR5 as a co-receptor (as opposed to CXCR4); however, these viruses are T-cell tropic (R5 T-tropic), not macrophage tropic (R5 M-tropic), requiring high densities of CD4 on the cell surface to mediate efficient entry. In contrast, M-tropic strains (able to enter cells efficiently with a low density of CD4) are typically found late in disease and compartmentalized, most often in the central nervous system (CNS). Sequencing and characterization of viral envelopes from rebound virus in the blood of individuals who are no longer undergoing cART demonstrated that all rebound viruses analyzed to date have been R5 T-tropic. Dr. Swanstrom concluded his talk by raising the issue of how relevant M-tropic strains are to the HIV reservoir.

Dr. Quentin Sattentau (University of Oxford, UK) next spoke on the “Spread of HIV-1 to and from macrophages.” He highlighted work characterizing cell-to-cell spread at the virological synapse, and the mechanisms of macrophage infection via phagocytosis of infected CD4^+^ T cells [11]. Interestingly, cell-to-cell infection through virological synapses of MDM and T cells were resistant to some BNAbs and antiretroviral drugs (raltegravir, nevirapine, and azidothymidine), due to infection dynamics and a high concentration of virus at the virological synapse. Together, this suggested that infected macrophages may contribute to reservoir persistence [12], disseminating infection despite cART, and that this might be limiting for future BNAb therapy.

Dr. Ronald Collman (University of Pennsylvania, PA) presented “Shaping the macrophage reservoir *in vivo*”. With his major focus on CD4-depleted, SIV-infected macaques, he highlighted work showing extensive macrophage infection in systemic and CNS compartments, and that the envelope glycoproteins of these viruses exhibited a CD4-triggered conformation, susceptible to CD4-induced epitope-specific antibodies (Ab). The CD4-depleted animals, devoid of T-cell helper function, lacked development of anti-SIV envelope Ab responses, allowing the viral envelope to adopt this CD4-triggered conformation and efficiently infect CD4-low macrophages. In CD4-depleted, HIV^+^ individuals with AIDS, infection of the brain and other macrophage rich tissues contributes to late stage pathology and morbidity [13].

Dr. Marcelo Kuroda (Tulane Primate Center, LA), gave the last talk of this session entitled “Long-lived macrophages and the HIV reservoir: lessons learned from the SIV macaque model”. Using BrdU to track the cell division of macrophages in the gut, he found that turnover of monocytes (which give rise to short-lived lamina propria and interstitial macrophages, CD163^+^CD206^-^) was associated with progression to AIDS, while the long-lived macrophages (submucosal and alveolar, CD163^+^CD206^+^) contributed to reservoir persistence and chronic inflammation. He proposed a mechanism whereby high monocyte turnover is associated with high levels of long-lived macrophage infection when cART is initiated during this high turnover phase [14]. Thus, he concluded that monocyte turnover not only predicted progression to AIDS, but also potentially predicted levels of long-lived macrophage infection.

Together, these presentations provided evidence to suggest that macrophages can be infected *in vivo*, and that certain circumstances (such as low CD4^+^ T-cell counts, CD8^+^ lymphocyte depletion, and high monocyte turnover) may potentiate higher levels of macrophage infection than others. Moreover, at least *in vitro* macrophage-based transmission of infection to CD4^+^ T cells (and *vice versa*) can be resistant to antiretroviral therapy and to some BNAbs, suggesting that current therapies may be insufficient or inadequate to stop cell-to-cell spread of the virus. Overall, the physiological relevance of macrophage infection and of M-tropic viruses is still open to discussion.

## SESSION #2: MACROPHAGES AND HIV PERSISTENCE, LATENCY, AND ACTIVATION

Dr. Christina Gavegnano, an assistant professor in Dr. Raymond Schinazi's group (Emory University, GA), opened the second session with her talk “The role of Jak/STAT signaling in myeloid-derived HIV reservoirs”. She discussed the use of Jak inhibitors to purge the reservoir. Specifically, the use of Ruxolitinib, an FDA-approved Jak1/2 inhibitor that can cross the blood brain barrier and can block HIV replication and associated inflammation in CD4^+^ T cells and macrophages. Showing that Jak inhibitors block MDM reservoir establishment, maintenance, and expansion *in vitro*, Dr. Gavegnano hypothesized that use of these inhibitors in HIV^+^ individuals might decrease the lifespan of the macrophage and CD4^+^ T-cell reservoir.

Dr. Mario Stevenson (University of Miami, FL) then discussed the “Role of macrophages in HIV-1 persistence under ART”, which addressed whether macrophages are a reservoir for HIV during cART. By determining the tropism in rebound viruses following ATI, Dr. Stevenson identified envelope proteins that were able to infect macrophages. In contrast to Dr. Swanstrom's work discussed in Session #1, M-tropic cell-free viruses were obtained from the serum of 5 out of 6 HIV^+^ patients, although these viruses only accounted for 3%–5% of all strains isolated from plasma. He emphasized the need to acquire post-ATI samples immediately, from the first viral RNA^+^ samples, as it is likely that any virus that grows out could evolve rapidly into a more T-tropic strain.

The following talk, “Capacity of Myeloid cells to Support SIV Replication *in vivo*”, was given by Dr. Jason Brenchley (NIH/NIAID, MD) and focused on characterizing the SIV reservoir in myeloid cells from SIVmac239 or SIVsmE660-infected macaque tissues. Replication competent viral DNA was found in macrophages of 37% of lymph nodes. In addition, splenic macrophages had viral DNA irrespective of whether or not the animals were treated with cART. In contrast to Dr. Swanstrom's work, Dr. Brenchley found no compartmentalization of SIV from macrophages (compared to CD4^+^ T cells from the same anatomical site). Additional human sampling revealed viral DNA in the broncheoalveolar lavage (BAL) macrophages from 1 of 10 cART treated individuals; however, this individual was not fully virologically suppressed (exhibited cART failure) [15]. Dr. Brenchley closed by commenting that engulfment of CD4^+^ T cells is still thought to be responsible for the majority of macrophage infections *in vivo*, because T-cell receptor DNA is found within infected myeloid cells.

Dr. Guido Poli (San Raffaele Scientific Institute, Italy) gave the final talk of the session, entitled “HIV-1 Restriction in M1-Polarized Human Macrophages: A Model of Reversible Latent Infection”. Similar to previous talks, he emphasized the difficulty of detecting *in vivo* infected macrophages during cART. Focusing on macrophage polarization towards traditional M1 (inflamma-tory) and M2 (reparative) phenotypes, he showed that polarization towards M1 suppresses HIV infection due to upregulation of the restriction factors, APOBEC3A, TRIM22, and CIITA. Interestingly, a second round of M1 polarization post infection (termed M1^2^) further restricted HIV replication [16]. However, viral replication from M1^2^ macrophages can be induced using allogeneic PHA-blast co-cultures, suggesting that M1^2^ polarization represents a potential *in vitro* model of reversible HIV-1 latency in primary macrophages.

In summary, the speakers presented work describing potential macrophage reservoirs *in vivo* and the detection of replication-competent viral DNA in macrophages from SIV-infected animals (Dr. Jason Brenchley). Multiple mechanisms, including direct infection and/or engulfment of infected CD4^+^ T cells by macrophages also may contribute to the infection of macrophages. Finally, methods that inhibit HIV replication in macrophages following successful infection together with cART to lower antigen load provide starting points for the investigation of potential treatments. Furthermore, methods that maximally stimulate macrophages to induce viral production can be used to quantitate the viral reservoir in humans and assess the efficacy of cure strategies.

**Figure 1.**
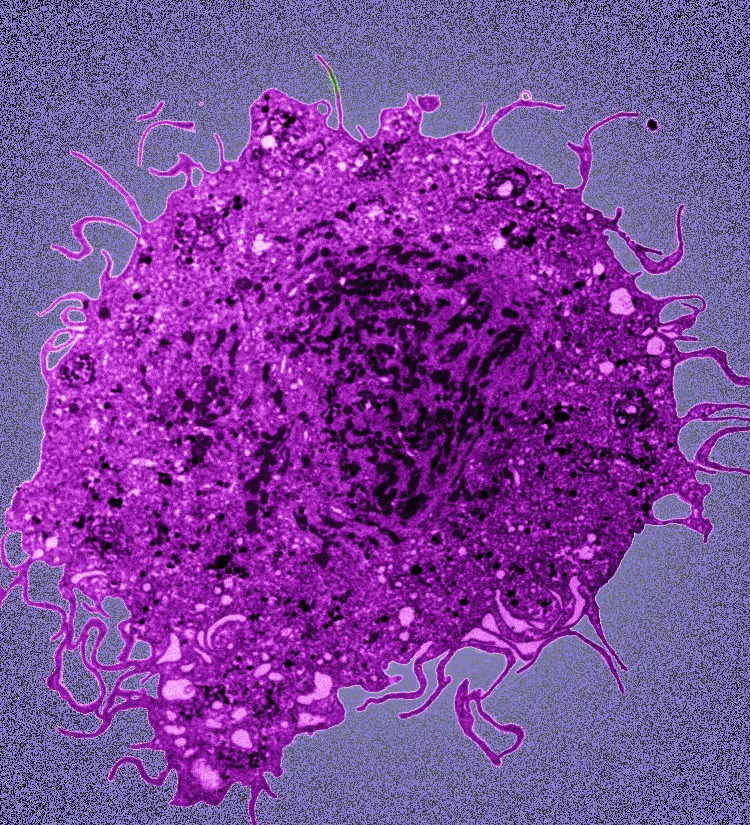
**HIV-infected macrophage.** Transmission electron microscopy picture of an HIV-infected macrophage taken by Dr. Mike Mashiba and Dr. Kathy Collins (University of Michigan).

## SESSION #3: HIV/SIV-INFECTED MACROPHAGES AND THE IMMUNE SYSTEM

Dr. Dionna Williams, a postdoctoral fellow in the laboratory of Dr. Janice Clements (Johns Hopkins University, MD), opened the third session with a talk entitled “Altered interferon-alpha regulation by infected macrophages”. She described earlier studies from their laboratory demonstrating delayed IFN-alpha signaling in the brain, compared to other compartments such as the lung, during acute SIV infection [17, 18]. Dr. Williams' studies demonstrated that the mechanism of altered IFN-alpha expression was due to astrocyte-derived CCL2, a chemokine that, on binding to CCR2, induced β-Arrestin activation that decreased IFN-alpha signaling in both uninfected and HIV-infected MDMs. These effects were more pronounced in HIV-infected versus uninfected macrophages and were specific to IFN-alpha, as no difference was observed for IFN-beta. While explaining the delay in CNS production of IFN-alpha, Dr. Williams' work also had implications for an initial reservoir establishment in the brain during acute infection due to defective type I IFN signaling.

Continuing with the theme of viral infection within the CNS compartment, Dr. Lishomwa Ndhlovu (University of Hawaii, HI) followed with his talk, “Insights into HIV reservoirs in the myeloid compartment with relevance to the CNS”. Employing HIV Gag p24 staining of the meninges and HIV RNA Scope staining of the cerebellum in brain tissues of cART-treated individuals, he showed that infection in the CNS presents many challenges for achieving an HIV cure. His studies also showed monocyte and macrophage inflammation in the brain, probably contributing to HIV-related neurological disorders and cognitive impairment. Dr. Ndhlovu's laboratory is currently studying epigenetic regulation in monocytes and the epigenetic differences that are indicative of CNS-related biological functions associated with cognitive impairment [19]. Understanding the changes in monocytes and their functions will provide insights for future therapies to address HIV-related neurological disorders.

The third talk of the session, entitled “CD8^+^ T-cell-mediated clearance of infected macrophages” by Dr. Kiera Clayton, a postdoctoral fellow in Dr. Bruce Walker's laboratory (Ragon Institute of MGH, MIT and Harvard), discussed the CD8^+^ T-cell response to infected macrophages versus infected CD4^+^ T cells. Compared to infected CD4^+^ T cells, HIV-infected macrophages were less susceptible to CD8^+^ T-cell-mediated cytoxicity, which agreed with previous reports in the SIV model by Dr. Stevenson and Dr. Watkins [20, 21]. This resistance is due to differential susceptibility to granzyme B, which is poorly co-expressed with perforin^+^ *ex vivo* CD8^+^ T cells, suggesting a potential mechanism for persistence of an HIV reservoir in macrophages. In addition, robust synapse formation with lack of killing induces strong CD8^+^ T cell IFN-γ responses, further inducing a 10-fold induction of macrophage-derived CXCL9 and CXCL10 chemokines that recruit CXCR3^+^CD4^+^ T cells. As was earlier described by Dr. Sattentau, interactions between infected macrophages and CD4^+^ T cells can result in efficient infection of CD4^+^ T cells. Thus, impaired macrophage killing and recruitment of HIV susceptible target cells could augment infection.

The final talk of the session by Dr. Kenneth Williams (Boston College, MA), entitled “Targeting monocyte and macrophage activation in SIV pathogenesis”, described in extensive detail the populations of brain macrophages involved in encephalitic lesion formation and the use of Mitoguazone (MGBG) to control inflammation. This S-adenosylmethionine decarboxylase inhibitor is selectively concentrated in macrophages via active transport and decreases HIV expression by inhibiting HIV integration in macrophages. Using SIV-infected macaques depleted of CD8^+^ T cells (a model of SIV encephalitis,), 50% of placebo controls developed SIV encephalitis (88% developed AIDS), while none of the MGBG-treated animals developed this disease (36% developed AIDS). Although there were no differences in plasma viremia or proviral DNA levels in monocytes or CD4^+^ T cells, MGBG decreased macrophage accumulation in the CNS and reduced the overall number of monocytes in the blood. In addition, the inhibitor decreased cardiovascular inflammation and fibrosis, also due to decreased macrophage accumulation.

Together, the studies discussed in this session described immune responses to HIV/SIV-infected macrophages. From an innate immunity perspective, inefficient type I IFN responses in the brain and in macrophages exposed to CCL2 during acute infection, could potentiate the establishment of a macrophage reservoir in the CNS and in other tissues. From an adaptive immunity perspective, impaired CD8^+^ T-cell macrophage killing could drive a persistent pro-inflammatory cytokine and chemokine response, thus contributing to recruitment of CD4^+^ T cells to sites of infection and chronic inflammation. Inflammatory monocytes and macrophages are very likely to contribute to HIV-related neurological disorders as well as to cardiovascular inflammation. Understanding these signatures of inflammation and using inhibitors, such as MGBG, to control HIV-associated inflammation will be essential for treating non-AIDS-related co-morbidities.

## SESSION #4: EMERGING TECHNOLOGIES FOR STUDYING HIV INFECTION IN MONOCYTES AND MACROPHAGES

Dr. Jenna Honeycutt, a postdoctoral fellow in Dr. J. Victor Garcia's laboratory (University of North Carolina at Chapel Hill, NC) spoke first about “Humanized mouse models for myeloid reservoir studies”. She described an elegant humanized mouse model lacking T cells and comprising only myeloid cells (the Myeloid only Mouse – MoM) [22]. Following CD34^+^ cell engraftment, the majority of the human myeloid cells, which develop into tissue macrophages including macrophages in the brain, are CD14^+^CD16^-^. These mice are susceptible to HIV-1 infection (shown for HIV-1 strains ADA and CH040) and maintain high viremia levels in peripheral blood (10^5^-10^6^ copies/mL) over many months. Furthermore, tissue sectioning showed HIV-infected macrophages (ie expressing viral RNA) in the liver, lung, and spleen. Finally, cART efficiently suppressed plasma viremia and reduced the HIV DNA and RNA in tissues. This work is a significant first step in the development of a small animal model for HIV-1 infection that offers unique access to HIV-infected tissue macrophages *in vivo*.

Dr. Jose Manuel Ordovas-Montanes, a postdoctoral fellow in Dr. Alex Shalek's laboratory (Ragon Institute of MGH, MIT and Harvard, Cambridge, MA), spoke about “Applying single-cell genomics to study tissue macrophages”. He described an RNA sequencing platform used to characterize macrophages directly from tissues with minimal manipulation. Using this single cell sequencing platform, Seq-well (an inexpensive high-throughput technology), transcriptomes of individual cells from small tissue biopsies (80,000^+^ cells) can be sequenced and analyzed. This technique will be an important contribution to future efforts to characterize tissue-resident macrophages in healthy and HIV^+^ individuals, providing insights into changes in macrophage differentiation and phenotype, and the frequency of infected macrophages in tissues.

Dr. Janice Clements (Johns Hopkins University, MD) gave the third talk of the session entitled “Quantitation of Functional Latent Macrophage Reservoirs in Tissues using a Viral Outgrowth Assay”. Her laboratory has adapted the traditional resting CD4^+^ T-cell-based quantitative virus outgrowth assay (QVOA) for HIV (developed by Dr. Robert Siliciano) for the SIV model, to quantify both resting CD4^+^ T cells and monocytes in blood as well as tissue CD4^+^ T cells and tissue-resident macrophages [23, 24]. Using CD11b expression to positively select cells, limiting dilution culture with CEMx174 cells and TNF activation was used to achieve viral outgrowth, which was quantified in the cell supernatant by RT-PCR. Confirmation of the absence of T cells in culture was confirmed by measuring T-cell receptor-beta RNA in wells without CEMx174 cells. Macrophage populations from multiple tissue compartments including blood, spleen, lung, brain, and BAL, were found to have varying frequencies of macrophages which were productively or latently infected in SIV-infected macaques treated with antiretroviral agents. Furthermore, the frequency of latent reservoirs of resting CD4^+^ T cells and macrophages in ARV-treated animals was similar in the blood and the spleen (~1 cell in 10^6^ cells assayed). This assay provided the first demonstration of latently infected macrophages that contained replication competent virus and provides a robust platform for future assessment of the macrophage viral reservoir in human tissues.

As the final talk for the session, Dr. Bjorn Corleis, a postdoctoral fellow in Dr. Douglas Kwon's laboratory (Ragon Institute of MGH, MIT and Harvard), discussed his work and that of Abby Schiff (Harvard Medical School MD/PhD candidate) on the detection of HIV-infected human macrophages entitled “Alveolar Macrophages as a Potential HIV Reservoir”. Alveolar macrophages isolated from the BAL of HIV^-^ and HIV^+^ (cART-naive) individuals were used for the isolation and detection of HIV DNA, RNA (by ddPCR), and integrated DNA (by Alu-PCR). These methods detected high levels of HIV RNA and DNA, but no integrated DNA in alveolar macrophages; however, all 3 viral products were found in CD4^+^ T cells. A fluorescence-based viral-entry assay was used to assess viral entry into alveolar macrophages, CD4^+^ T cells from PBMCs and BAL, and MDMs; results showed that viral entry was least efficient in the alveolar macrophages, likely due to low expression of CD4 and CCR5. These results suggested that alveolar macrophages did not significantly contribute to productive infection in the chronic phase of infection. This work stresses the importance of using Alu-PCR in future studies to specifically detect integrated DNA as opposed to total DNA, which could be derived from infected, engulfed CD4^+^ T cells.

Overall, significant advances in technology development, including the macrophage QVOA, high-throughput single-cell sequencing, and techniques to simultaneously assess multiple HIV products, provide a framework to characterize the macrophage reservoir in humans. The remaining challenges are access to human tissues and the biases inherent to having access to only a fraction of the tissues that might be infected, most importantly the brain. The MoM model of HIV infection and SIV-infected macaques are essential tools not only to study tissue-resident macrophages, but also to develop future technologies to address the challenges currently faced by those studying cells in HIV^+^ individuals.

## CONCLUDING SESSION: SETTING RESEARCH PRIORITIES

Concluding the meeting was a session led by Dr. Dan Kuritzkes (Brigham and Women's Hospital, MA), Dr. Jeymohan Joseph (NIMH, MD) and Dr. Diane Lawrence (NIH/NIAID, MD), with contributions from Dr. Jim Demarest (ViiV Healthcare). Together, they provided perspectives on the research presented and the questions that will need to be addressed to confirm the importance of macrophages to the viral reservoir and disease pathogenesis, and how this relates to the development of strategies for a cure. Five topics were discussed: (1) biology, (2) pathogenesis, (3) reservoir, (4) eradication, and (5) model systems.

For the topic of macrophage biology, the focus was on the establishment of HIV infection and latency in macrophages. While much is known about latency in CD4^+^ T cells, the question remains whether HIV establishes a truly latent and persistent reservoir in macrophages. The following questions were presented:

- Is HIV infection/latency in macrophages different in different tissues?
- Is HIV infection/latency different for MDM versus embryonically derived macrophages?
- How is viral transcription regulated in infected macrophages?
- Can the macrophage reservoir reseed the T-cell reservoir?

One major difficulty in answering these questions from a human immunology setting is the availability of appropriate samples. However, given that HIV^+^ donor to HIV^+^ recipient transplants are now approved in certain states, this may be a future source of more comprehensive tissue samples for research. In addition, access to tissues post-autopsy is also a source of tissues from HIV^+^ individuals and should be explored.

In terms of the contribution of macrophages to HIV pathogenesis, multiple presentations at this meeting confirmed, in animal models, that chronic activation of monocytes and macrophages drives SIV/HIV-associated neurological disorders, encephalitis, and cardiovascular inflammation. Although treatment with MGBG can be used to combat this inflammation, these questions still remain:

- What is the effect of cART on persistent inflammation in HIV^+^ individuals?
- How does residual viremia contribute to this inflammation?
- If monocytes and macrophages are at the center of inflammation pathways, what therapies can be used in combination with cART to address this issue?

The panelists agreed that more pathogenesis research is needed to fully understand the contribution of monocytes and macrophages to inflammation and should be addressed when considering potential strategies for cure.

For the next topic of discussion, the HIV/SIV reservoir was the major focus. Some of the presentations at this meeting provided evidence that macrophages are infected in both SIV-infected macaques, HIV-infected humans, and humanized mice models and can sustain productive infection. However, the full contribution of macrophage infection to the establishment and maintenance of the latent reservoir in human infection, especially in the presence of cART, is still open for debate. Questions that remain include the following:

- Do macrophages directly or indirectly contribute to the latent and/or persistent viral reservoir?
- How do HIV sequences in macrophage populations from different tissues relate to each other and to plasma virus?
- What are the methods to identify different subpopulations of HIV-infected macrophages and their infection profile?
- Within HIV-infected cART-treated individuals, do short-lived MDMs or long-lived embryonically derived macrophages contribute most to the reservoir and persistent innate activation?

Again, current tissue sampling is a major barrier to answering these questions, but techniques such as single cell RNA sequencing and the macrophage QVOA will help to combat this issue.

When addressing HIV eradication, investigations of macrophage reactivation to induce expression of viral proteins are in their infancy. Furthermore, caution is needed when considering reactivation of macrophages in the brain because this could cause excess immune activation and encephalitis. The following questions were considered:

- How can infected macrophages be targeted by the immune system?
- How will the impact of interventional studies on macrophage infection be assessed and quantified?

Following discussion of these questions, a controversial suggestion was offered: should we “let sleeping dogs lie”? In other words, should all infection be eradicated or would a functional cure in combination with therapies to combat inflammation be sufficient for HIV^+^ individuals?

Model systems and improvement of the current systems were the final topics addressed. The SIV/ macaque model and the HIV/humanized mouse model have provided much insight into the contributions of macrophages to infection and will serve as the most appropriate models to test interventional strategies. However, these additional refinements are needed:

- the determination of the type of tissue macrophages present in humanized mice
- further sampling of the SIV-infected macaque brain
- the development of organoid cultures to better assess human macrophage samples *in vitro*

Addressing these issues will help us better understand basic macrophage biology and how this affects the establishment and maintenance of the viral reservoir and disease pathogenesis.

Ultimately, the discussions in this workshop provided insights for guiding research priorities to be pursued by NIH OAR, NIMH, the NIAID, and industry partners. The participants of the workshop look forward to pursing the topics discussed during the meeting and attending a subsequent meeting within the next 2 years. This gathering and contributions by experts in the fields of HIV/ SIV immunology and virology, macrophage immunology and biology, and animal models of HIV infection will lead to answers for these important questions and ultimately to achieving an HIV cure.
